# UVC Stokes and Anti-Stokes Emission of Ca_9_Y(PO_4_)_7_ Polycrystals Doped with Pr^3+^ Ions

**DOI:** 10.3390/molecules29092084

**Published:** 2024-05-01

**Authors:** Karol Lemański, Olha Bezkrovna, Nadiia Rebrova, Radosław Lisiecki, Patrycja Zdeb, Przemysław Jacek Dereń

**Affiliations:** 1 Institute of Low Temperature and Structure Research, Polish Academy of Sciences, ul. Okólna 2, 50-422 Wrocław, Polandn.rebrova@intibs.pl (N.R.); r.lisiecki@intibs.pl (R.L.); p.zdeb@intibs.pl (P.Z.); 2 Institute for Single Crystals, NAS of Ukraine, Nauky Ave. 60, 61001 Kharkiv, Ukraine

**Keywords:** spectroscopy, phosphates, praseodymium, UVC upconversion emission, polycrystals

## Abstract

The recent COVID-19 pandemic has made everyone aware of the threat of viruses and the growing number of antibiotic-resistant bacteria. It has become necessary to find new methods to combat these hazards. One tool that could be used is UVC radiation, i.e., 100–280 nm. Currently, the available sources of this light are mercury vapor lamps. However, the modern world requires more compact, mercury-free, and less energy-consuming light sources. This work presents the results of our research on a new material in which efficient UVC radiation was obtained. Here, we present the results of research on Ca_9_Y(PO_4_)_7_ polycrystals doped with Pr^3+^ ions prepared using the solid-state method. The absorption, excitation, emission, and emission decay profiles of praseodymium(III) ions were measured and analyzed. The upconversion emission in the UVC region excited by blue light was observed. Parameters such as energy bandgap, refractive index, and thermal stability of luminescence were determined. The studied phosphate-based phosphor possesses promising characteristics that show its potential in luminescent applications in future use in medicine or for surface disinfection.

## 1. Introduction

UVC radiation (100–280 nm) can successfully inactivate microbes and viruses [1], better than UVB (280–315 nm) or UVA (315–400 nm) light. Phosphors capable of emitting light in the UVC range could have a wide range of medical applications, from self-cleaning surfaces to various medical procedures [2,3,4,5], e.g., to sterilize surgical equipment, room disinfection as well as air and water, which is due to the fact that UV sterilization helps to eliminate dangerous pathogens in many public places, e.g., hospitals, workplaces, schools, or airports.

An important issue for obtaining UVC radiation is the choice of host matrix and activator. Rare-earth elements are most often used as an activator. Among them, Pr^3+^ has attracted much attention. Praseodymium(III) has two electrons in the 4f subshell, well shielded by other subshells from the influence of the crystal field, which is characteristic of all lanthanides (Ln). Because of this, the lanthanides in various crystalline compounds possess stable spectroscopic properties. The luminescence of Pr^3+^ ions can be characterized by many colors from near-ultraviolet and blue to infrared range [6,7,8,9,10,11,12,13,14]. Most often, blue or red emissions dominate, which mainly occurs, respectively, from the ^3^P_0_ or ^1^D_2_ energy levels to the ground state ^3^H_4_. Also, in some hosts doped with Pr^3+^ ions, a strong 5d→4f emission in the UVC range is observed [1,15,16,17].

In recent years, the accommodation of rare-earth elements in phosphate-based hosts has been widely studied in connection with the luminescent properties they exhibit. The crystallographic properties of phosphates favor this doping. Phosphates have a lot of applications [18,19,20]. They are also widely used in the healthcare industry. These compounds are present naturally in human teeth and bones, and they are used in the manufacturing of medicines for these organs [21,22,23]. They are also used in toothpaste as a polishing agent and to enable the free flow of the paste through a tube. Another important characteristic of phosphates is their prevalence in living organisms. Calcium phosphates are also added to animal feed [20,24,25]. Therefore, we hope that future biocompatibility testing of our best phosphate material will demonstrate its potential for medical applications [19,26].

Calcium phosphate Ca_9_Y(PO_4_)_7_ is also a potentially good matrix for doping lanthanide ions in it to obtain efficient luminescence [27,28,29,30,31]; however, the available scientific literature on this matrix is still quite modest. This material is well suited for doping with lanthanide ions, e.g., because the trivalent lanthanide ions are well matched in structure to yttrium ions in terms of good ionic size and valence match. Ca_9_Y(PO_4_)_7_ also has a sufficiently large energy gap and an appropriate position of the 5d level that allows Stokes and anti-Stokes emission of Pr^3+^ ions to occur.

In the context of the UVC upconversion, phosphates, despite their high phonon energies [32], which increase the nonradiative multi-phonon relaxation (MPR) rate, can be chosen for the study due to two main advantages. First of all, they possess a wide bandgap that allows for UVC emissions to be observed. Second, some rare-earth doped phosphates also exhibit a significant UV/VIS branching ratio, which can be attributed to small shifts of the parabolas of the 5d vs.4f electronic configuration [17].

The objective of our work was to test the ability of Pr^3+^-doped Ca_9_Y(PO_4_)_7_ phosphate (CYPO) to demonstrate UVC upconversion emission when excited by a blue light. The structure of Ca_9_Y(PO_4_)_7_ is a derivative of the structure of Ca_3_(PO_4_)_2_. The prevalence of calcium phosphates in the human body makes the derivatives of Ca_3_(PO_4_)_2_ highly biocompatible. The undoped CYPO matrix does not exhibit intrinsic luminescence, which could interfere with the luminescence of possible rare-earth activator ions. Thus, Ca_9_Y(PO_4_)_7_ doped with Pr^3+^ ions is a promising candidate to obtain the emission in the UVC range induced by the upconversion processes. This feature can later be used in the production of UVC LEDs to destroy viruses and bacteria. The obtained phosphors possess remarkable luminescent properties and high chemical stability and are relatively cheap.

Similar matrices of calcium phosphates were investigated with several doped lanthanides like Eu^3+^, Dy^3+^, Pr^3+^, and Gd^3+^ ions [16,17,27,31,33,34,35,36,37]. These compounds possess interesting crystal structures and, thus, are often investigated by doping lanthanide ions, which exhibit various spectroscopic properties, which mainly depend on the different dopants and other factors, like crystal symmetry, temperature, or pressure. Among the suggested applications, Ca_9_Y(PO_4_)_7_ doped with Eu^3+^, Tm^3+^, and Dy^3+^ may be promising candidates for light-emitting diodes [27,29]. Additionally, the Ca_9_Y(PO_4_)_7_:Tm^3+^,Yb^3+^ phosphor has potential application as an efficient luminescent thermometer in the temperature range of 323–823 K [30]. Based on the structure of Ca_3_(PO_4_)_2_, the structure of these phosphates was also studied with the direct entry of doped rare-earth ions into the structure as Ca_9_RE(PO_4_)_7_ (where RE is a rare-earth ion, in this case, Nd, Gd, or Dy) [38].

F. Piccinelli et al. investigated a similar whitlockite-like crystal structure of Ca_9_Lu(PO_4_)_7_ phosphate, also doped with Pr^3+^ ions. In this case, four crystal sites can be occupied by RE^3+^ ions, but, e.g., Eu^3+^ or Pr^3+^ doped ions prefer to occupy two and three sites, respectively [35]. Camardello et al. studied Ca_9_R^3+^(PO_4_)_7_ (R^3+^ = Al, Ga, Sc, Lu, Y, Gd, La) phosphates doped with Ce^3+^ and Pr^3+^ ions [17]. They discovered that the optical properties are independent of the RE^3+^ cations, which was connected with the preferential occupation of the eight-coordinated calcium site. Chien-Hao Huang et al. showed the UV luminescence of Ca_9_Y(PO_4_)_7,_ while doped with 0.2% Pr^3+^, under excitation at 172 nm. In this work, the authors claim that Pr^3+^ ions are expected to occupy the Y^3+^ ions sites [28].

Despite several works describing the properties of Ca_9_Y(PO_4_)_7_:Pr^3+^, none of them present the luminescent analysis of Ca_9_Y(PO_4_)_7_ for various concentrations of doped praseodymium in such a broad way. In this work, we present the spectroscopic study of Ca_9_Y(PO_4_)_7_ polycrystals doped with Pr^3+^ ions, with the results of the UVC upconversion emission discovered for the first time in this crystal host.

The investigated phosphors are suitable as materials that are more compact and less energy-consuming light sources because they are polycrystalline powder materials; thus, a small amount of them is enough to obtain the appropriate effect. Moreover, the fact that these materials exhibit luminescence in the UVC range, not only through Stokes emission but also through anti-Stokes emission, significantly reduces the potential costs of such a device, as this emission could be excited by radiation in the visible range matched to the wavelength of commonly used blue LEDs.

## 2. Results and Discussion

### 2.1. Structure Analysis

**Figure 1:**
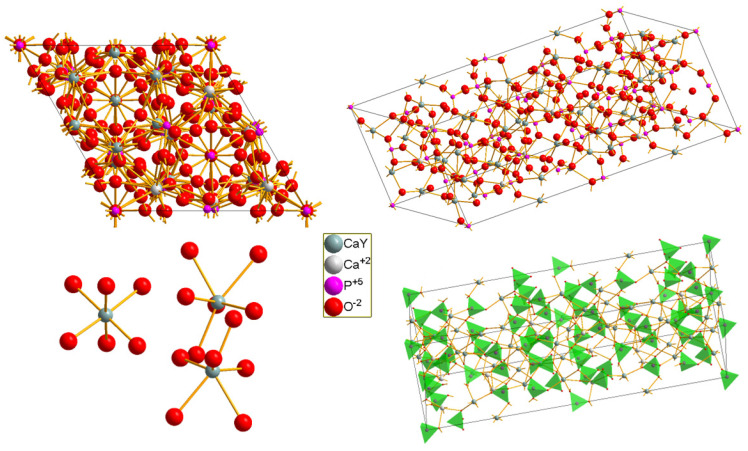
Visualization of the Ca_9_Y(PO_4_)_7_ crystal structure.

Calcium yttrium heptaphosphate Ca_9_Y(PO_4_)_7_ (ICSD: 236034) possesses a crystallographic structure in which there are as many as seven crystallographic positions (sites) for cations, four for Ca^2+^ ions and three for Y^3+^. The space group is trigonal *R3c* (161). The cell parameters are a = 10.4400(5) Å c = 37.3646(4) Å, a/b = 1.0000 b/c = 0.2794 c/a = 3.5790; V = 3526.89(34) Å^3^ and Z = 6 [39]. Due to the presence of yttrium (Y^3+^) ions, which both in terms of charge matching and ionic size are similar to doped lanthanide ions, they are located in the crystal structure without creating defects compensating for the electric charge. The visualization of the unit cell of the measured crystal is shown in Figure 1.

Calcium(II) ions in the CYPO crystal structure can be found in four sites, while yttrium(III) ions, and praseodymium ions, can be found in three crystallographic positions. These positions are shared with calcium ions. Detailed metal–ligand distance data can be found in Table S1. There are four crystallographic positions for calcium in the investigated structure, three of which are related to the positions of yttrium. Large Pr^3+^ ions should preferentially occupy the eight-coordinated site in this structure [17]. Y^3+^ ions are located in three crystallographic positions, two with eight coordinations, which have metal–oxygen distances very close to each other, and one position with a coordination of six. Therefore, the doped Pr^3+^ ions would mainly occupy the Y1 and Y2 sites which are coordinated by eight oxygen ligands.

**Figure 2:**
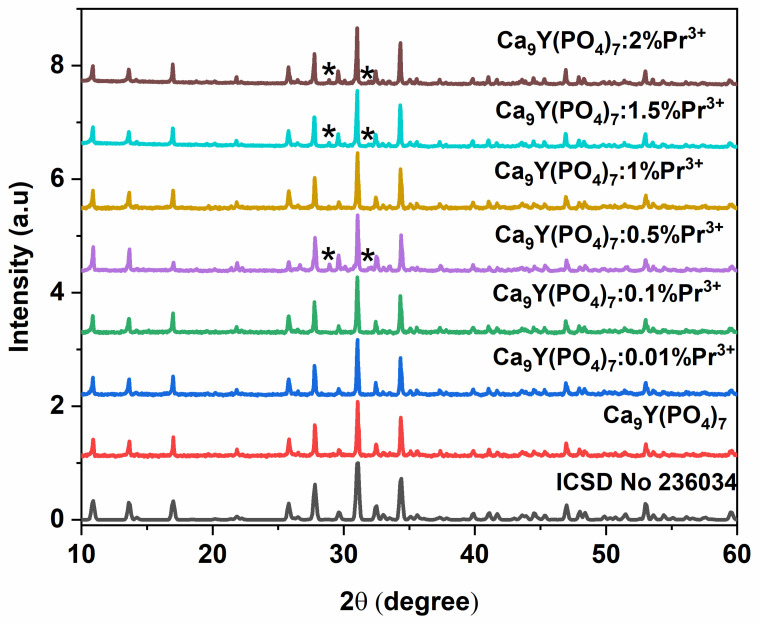
X-ray diffraction patterns of Ca_9_Y_1-x_Pr_x_(PO_4_)_7_:Pr^3+^ particles and standard reference of Ca_9_Y(PO_4_)_7_. Y_2_O_3_ impurity reflections are shown by asterisks.

Figure 2 shows the XRD spectra of pure and Pr^3+^ doped CYPO. All patterns could be indexed based on the trigonal structure SG: *R3cH* (ICSD No. 236034). Some reflections in the XRD patterns belong to the Y_2_O_3_ (ICSD 96-720-5918) impurity and are marked in Figure 2 by asterisks. The calculated lattice parameters of the samples are presented in Table 1. The results showed that the host lattice slightly expands with increasing activator concentration in the nanoparticles (see Table 1) because the ionic radii of Pr^3+^ are larger than that of Y^3+^ [40].

According to the diffraction data, the size of the crystallites (*D*) was estimated using the Scherrer equation [41]:

*D* = *Kλ*/*β* cos*θ* (1)

where *K* is the shape constant equal to 0.9, *λ* is the wavelength of the incident X-ray (1.54056 Å), *θ* is the Bragg angle, and *β* is the Full Width at Half Maximum (FWHM) of the diffraction peak. The crystallite size of the Ca_9_Y_1-x_Pr_x_(PO_4_)_7_ samples, estimated by the Scherrer formula, does not depend on the concentration of the activator (Table 1). Since the crystallite size can be affected by lattice deformation and lattice defects, the size and the strain were estimated using the Williamson–Hall (W–H) plot method [42,43] according to the following formula:

(2) $$\beta hkl*\cos\theta =\frac{k\lambda }{D}+4\varepsilon *\sin\theta$$

where *ε* is the strain. Linear fitting of dates obtained by plotting *β*cos*θ* (x-axis) and 4*ε*sin*θ* (y-axis) was used to estimate crystallite size from y-axis intersections and strain from slope (Figure S1). The strains and the crystallite size values are included in Table 1. Measurements by the W–H methods revealed that the crystallite size of the powders increases continuously with increasing Pr^3+^ concentration. It should be noted that the crystallite sizes calculated using the Scherrer method are somewhat smaller than those calculated from the W–H plot method. This can be explained by the fact that the Scherrer method does not take into account any particle agglomerations.

### 2.2. Optical Characterization

The refractive index of CYPO crystal together with the energy bandgap E_g_, was estimated using the method based on the chemical formula [44,45]. Calculated results are as follows: the refractive index *n* = 1.67 and E_g_ between 6.22 and 6.55 eV. The obtained relatively high value of E_g_ makes this crystal suitable for applications, especially as a phosphor in the UV range.

Absorption spectra of the investigated polycrystals doped with Pr^3+^ ions show the absorption peaks characteristic of the used dopants. Figure S2 shows transitions from the ground ^3^H_4_ energy level to the next excited states of the praseodymium(III). The center of gravity of the ^1^D_2_ absorption level is at 596 nm (16,779 cm^−1^), with the whole band ranging from about 570 to 620 nm, with a maximum observed at 591 nm (16,920 cm^−1^).

### 2.3. Phonon Properties

The primitive unit cell of the Ca_9_Y(PO_4_)_7_ *R3c* structure is composed of 90 atoms having 270 degrees of freedom (45A_1_ + 45A_2_ + 90E). According to the factor group analysis, 267 of them (45A_1_ + 45A_2_ + 90E) are optical modes, whereas 3 are acoustic ones (A_1_ + E). Because the 45A_2_ modes are silent and the structure is polar, the remaining 44A_1_ and 89E symmetry modes are both IR- and Raman-active. As a result, 133 IR and Raman bands are expected.

**Figure 3:**
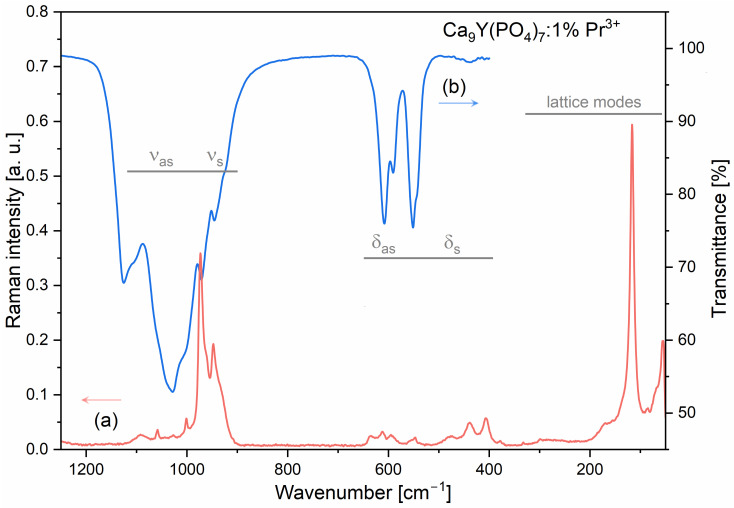
Raman (**a**) and IR (**b**) spectra of the Ca_9_Y(PO_4_)_7_ sample doped with 1 mol% of Pr^3+^ ions.

Figure 3 demonstrates the IR transmittance and Raman spectra measured for the representative sample containing 1 mol% of Pr^3+^ ions. The registered spectra are very similar to previously reported data for the Ca_9_Y(PO_4_)_7_ *R3c* structure and other similar compounds, i.e., Ca_9_Bi(PO_4_)_7_ [46], Ca_9_RE(PO_4_)_7_ (RE = La, Pr, Nd, Eu, Gd, Dy, Tm, Yb) [47], and Ca_9_Y(PO_4_)_7(1−x)_(VO_4_)_7x_ [48]. The Raman spectrum of undoped Ca_9_Y(PO_4_)_7_, previously reported by Zhang et al., shows the presence of Raman bands at 404, 440, 610, 945, and 971 cm^−1^ [48]. Our studies indicate that the corresponding Raman bands are upshifted by 1–3 cm^−1^, which can be attributed to different methods of synthesis and/or the presence of heavier Pr^3+^ ions. The IR spectrum of Ca_9_Y(PO_4_)_7_, to the best of our knowledge, has not been reported so far.

All the observed IR and Raman bands can be divided into internal vibrations associated with phosphate ions and lattice modes. Based on the literature data [47,49,50], they were assigned as follows: 377–557 cm^−1^ to the symmetric bending modes (δ_a_), 590–636 cm^−1^ to the antisymmetric bending modes (δ_as_), 925–973 cm^−1^ to the symmetric stretching modes (ν_s_), and 1001–1125 cm^−1^ to the antisymmetric stretching modes (δ_as_) of the deformed tetrahedral phosphate ions. All Raman bands below 332 cm^−1^ were assigned to the lattice modes, which included the translational and vibrational modes of orthophosphate ions, as well as translations of Ca^2+^ and Y^3+^/Pr^3+^.

### 2.4. Emission in the UVC Range

**Figure 4:**
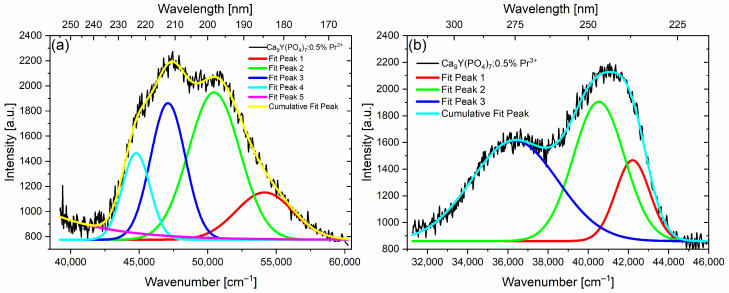
The room temperature excitation (**a**) and emission (**b**) spectra of CYPO:0.5% Pr^3+^ polycrystals. The spectra are fitted to a sum of Gaussian bands.

Measurements in the UVC range were carried out using a deuterium lamp and the McPherson spectrometer. Figure 4 and Figure S3 present the excitation and emission spectra of CYPO:0.5%Pr^3+^ polycrystals, which correspond to the 4f^2^↔4f^1^5d^1^ interconfigurational optical transitions.

The excitation band monitored at 275 nm decomposed into the sum of Gaussian bands centered at 54,146 cm^−1^, 50,473 cm^−1^, 47,103 cm^−1^, and 44,782 cm^−1^ (Figure 4a). The emission spectra excited at 196 nm were deconvoluted into Gaussian components centered at 42,221 cm^−1^, 40,532 cm^−1^, and 36,331 cm^−1^ (Figure 4b). Based on the excitation and emission spectra, the Stokes shift (ΔS) was estimated by comparing the position of the lowest energy absorption maxima and the position of the highest energy emission maxima. The Stokes shift calculated for CYPO:0.5% Pr^3+^ polycrystals is 2561 cm^−1^. Notably, this value is nearly 1000 cm^−1^ larger than the Stokes shift reported for the Ca_9_Y(PO_4_)_7_:1% Pr^3+^ sample in the work of [17]. This is mainly caused by differences in the excitation spectrum, which for that publication is slightly wider in the lower energy range. The observed difference may be due to the differences in the sample synthesis and spectrophotometer sensitivity. Another explanation may be due to different crystallite sizes, given the difference in annealing temperatures between samples. According to this [17] work, efficient Pr^3+^ interconfigurational emission transitions of this compound may also be due to the fact that the Stokes shift is (like in our case) less than about 3300 cm^−1^, the 4f^1^5d^1^-4f^2^ radiative transition dominates over the nonradiative relaxation from the 4f^1^5d^1^ to the 4f^2^ levels of Pr^3+^ ions [16,17].

Interconfigurational transitions are allowed by Laporte’s selection rule [51,52,53] and in this case, in the comparison to the result of the luminescence decay times, the luminescence intensity increases with the addition of Pr^3+^, so that the number of emission centers responsible for this luminescence increases. The obtained emission in the UVC range is well matched to the radiation, which is characterized by disinfecting properties, eliminating viruses and bacteria.

**Figure 5:**
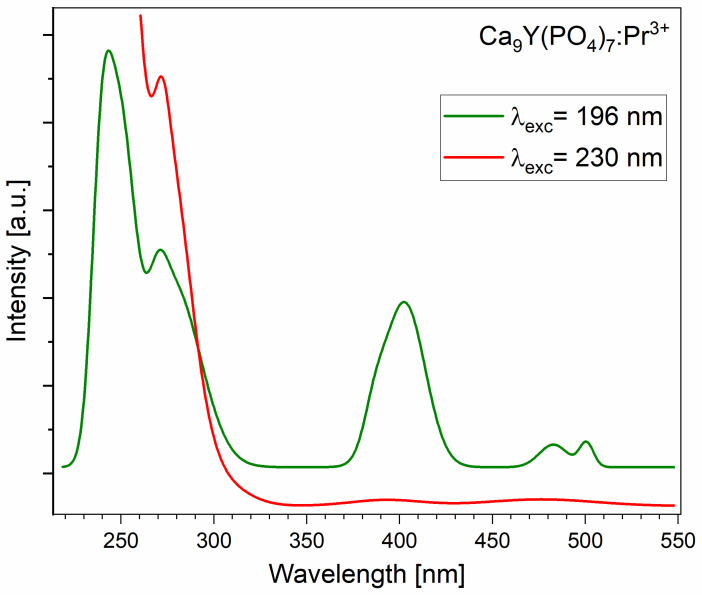
The UVC emission spectrum of Ca_9_Y_1-x_Pr_x_(PO_4_)_7_ polycrystals, at 300 K.

Figure 5 reveals the presence of at least two distinct Pr^3+^ sites. Excitation of Ca_9_Y_0.995_ Pr_0.005_(PO_4_)_7_ at shorter wavelengths, specifically 196 nm, activates all Pr^3+^ ions in the host, while excitation of Ca_9_Y_0.99_ Pr_0.01_(PO_4_)_7_ at 230 nm selectively activates only one class of Pr^3+^ ions located at the site with a stronger crystal field. The spectrum obtained with the former excitation demonstrates not only strong 4f^1^5d^1^ → ^3^H_J_ + ^3^F_2,3_ transitions observed between 220 and 320 nm, but also relatively strong 4f^1^5d^1^ → ^3^P_J_ transition with the peak at 403 nm. Since the ^3^P_J_ term is radiatively excited, transitions to ^3^H_4_ from ^3^P_0_ and ^3^P_1_ are also observed. However, under 230 nm excitation, the 4f→4f emission is notably weak, indicating minimal energy transfer from the 5d to 4f electronic configuration. This characteristic is attributed to the low value of the Stokes shift [17], which in our case amounts to 2561 cm^−1^.

**Figure 6:**
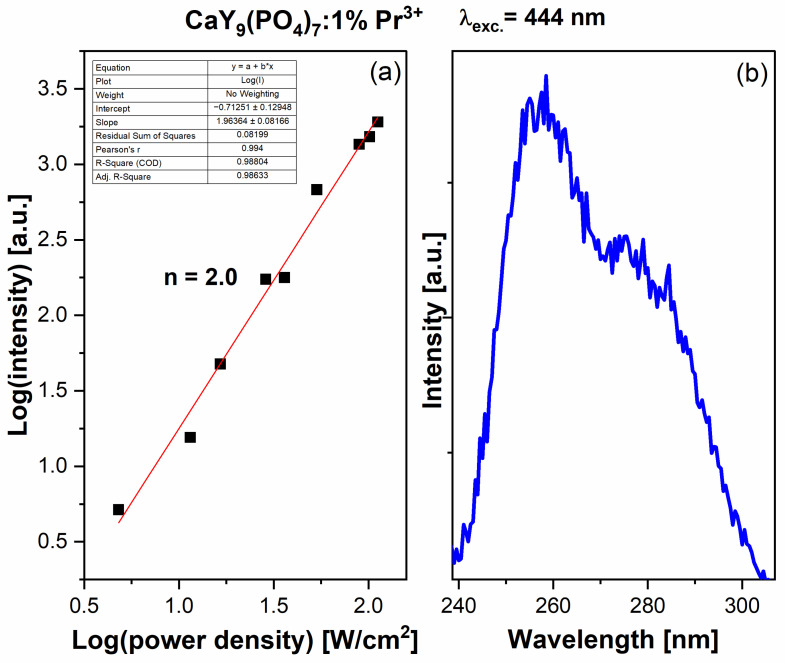
(**a**) Power density (per unit area) dependence of the upconversion emission of Ca_9_Y_0.99_Pr_0.01_(PO_4_)_7_ polycrystals; (**b**) The anti-Stokes emission spectra of Ca_9_Y_0.99_Pr_0.01_(PO_4_)_7_ polycrystals in the UVC range.

Since the 5d bands are within the range of blue light excitation, upconversion into ultraviolet radiation was observed. The influence of the excitation power “P” of the diode laser (444 nm) on the luminescence intensity “I” of the 5d-4f band in the UVC range was investigated. Detailed data are marked in Figure 6a. The characteristic in the log/log scale is linear and is proportional to I ~ P^n^, where *n* is the number of pump photons required to excite the emitting level [54]. The obtained result of *n* = 2 suggests that two photons are involved in the upconversion process. Figure 6b presents this anti-Stokes emission spectrum, with laser excitation at 444 nm. The results obtained after integrating the emission area show that most (about 72%) of the obtained UV upconversion emission is in the UVC range. The UVB part, which is less harmful to the viruses, possesses only 28% of the area in the emission spectrum.

**Figure 7:**
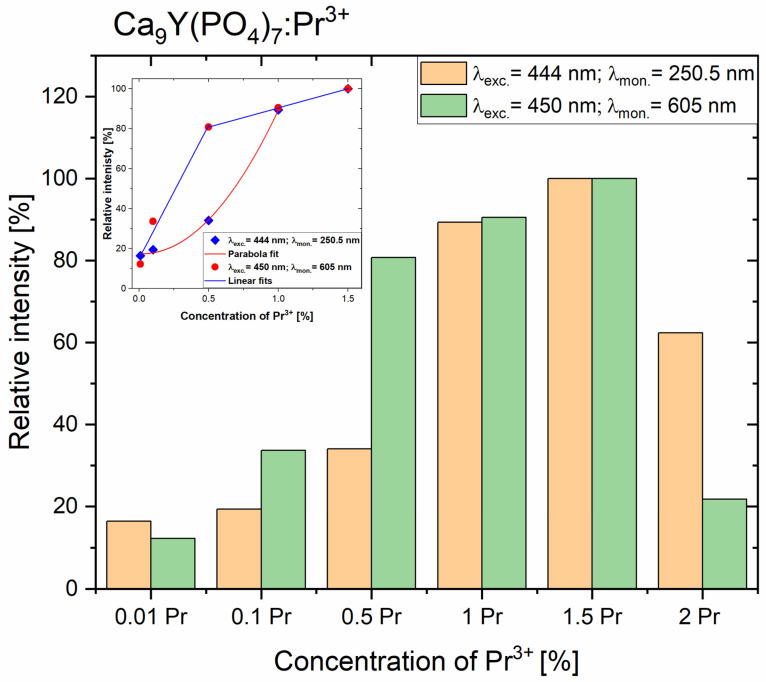
Relative intensities of the UVC upconversion and Stokes emissions of Ca_9_Y(PO_4_)_7_ polycrystals, for different concentrations of the doped Pr^3+^ ions. Inset shows the fitted intensity of these emissions.

For the CYPO powders studied, measurements of luminescence intensity “I” of upconversion and Stokes emissions were performed as a function of Pr^3+^ concentration “X” (see Figure 7). The intensity of emission could be approximated with such a function I ~X^a^, where “a” is the value of an exponent. For initial Pr^3+^ concentrations (for which so-called concentration quenching does not occur), the UVC upconversion emission was fitted with a quadratic function, while the Stokes emission depends linearly on the Pr^3+^ concentration there (see inset in Figure 7). The quadratic dependence of UVC emission intensity on Pr^3+^ concentration indicates the process of energy transfer upconversion (ETU) [54], which occurs in pairs of Pr^3+^ ions. Please note that for low concentrations, e.g., 0.5% Pr^3+^ the intensity of upconversion emission is relatively low compared to Stokes luminescence. On the other hand, a sample with a praseodymium ion concentration of 2% has a much weaker Stokes emission (about 20% compared to the most intense sample), while the anti-Stokes emission in the UVC region, although weaker, possesses a 60% intensity of the best CYPO sample (see Figure 7).

### 2.5. Spectroscopic Properties in the Visible Range

**Figure 8:**
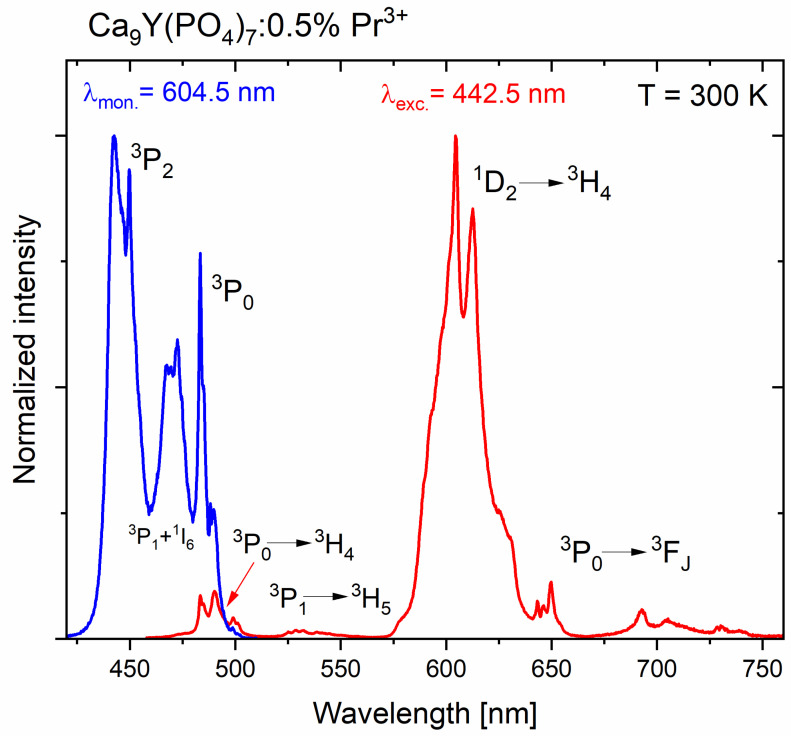
Excitation and emission spectra of Ca_9_Y_0.995_Pr_0.005_(PO_4_)_7_ polycrystals.

Figure 8 shows both, the excitation and luminescence spectra of CYPO polycrystals doped with Pr^3+^ ions. The most intense emission comes from the transition from level ^1^D_2_. The excitation spectrum in the UVC range, when monitoring the ^1^D_2_ → ^3^H_4_ transition, was very weak. Three peaks from the transition ^3^P_0_ → ^3^H_4_ are observed. The most intense is the red luminescence ^1^D_2_ → ^3^H_4_ and for it, the greatest influence of temperature change is observed. A small emission peak at 530 nm comes from a transition from the ^3^P_1_ level, because this level can be populated at room temperature with electrons from the neighboring ^3^P_0_ emitting level. The shape of the excitation and luminescence bands is like the emission of Pr^3+^ ions in similar calcium phosphates, where instead of yttrium (as in this case), aluminum and lutetium ions were present in the crystal matrix [34].

The mechanisms of excitation, luminescence, as well as concentration quenching by cross-relaxation (CR) are as follows. When excitation by a laser diode or xenon lamp by energy matched to the ^3^P_2_ praseodymium level, non-radiative transfer of the excitation energy to the ^3^P_0_ and ^1^D_2_ emitting levels occurs. Then, the emission from these levels to the ground state ^3^H_4_ and higher excited states take place. The observed luminescence is also quenched by a non-radiative cross-relaxation process that occurs in ion pairs when ions are close enough to each other according to the following schemes: [^1^D_2_, ^3^H_4_] → [^1^G_4_, ^3^F_4_]. When excited in the ^3^P_2_ band, the energy is transferred non-radiatively to the nearby ^3^P_0_ level, from which luminescence occurs. On the other hand, the emission from the ^1^D_2_ level can be populated from the ^3^P_0_ level by multi-phonon relaxation due to the high energy of the phonons in the phosphate matrices of around 1200 cm^−1^ [32].

The energy bandgap law states that if the difference between two energy levels is greater than five times the value of the highest energy phonon in the host medium, the probability of multiphonon relaxation will be negligible. The multiphonon non-radiative transition rates “*W_NR_*” can be written using the formula [55,56]:

*W_NR_* = (1/*t*_0_)exp(-*ap*), (3)

where *p* = ∆E/*hv*_max_ is the number of phonons needed to overcome the ∆*E* gap, *h* is the Plank constant, *v*_max_ is the highest phonon frequency in the host, and *t*_0_ and *a* are the empirically fitted parameters. The absorption spectrum shows that the energy distance between the ^3^P_0_ and ^1^D_2_ levels is less than 4000 cm^−1^; therefore, in the case of this host as well as other phosphates, since *p* ≈ 3, the probability of multiphonon relaxation will be significant. The cross-relaxation process [^3^P_0_, ^3^H_4_] → [^3^H_6_, ^1^D_2_] could also populate the ^1^D_2_ level but is much weaker than those from ^1^D_2_ because, according to the selection rule, they are spin-forbidden.

The luminescence intensity of the 4f emission decreases with increasing temperature, but it is a slow decrease and stable in the range of temperatures measured up to 540 °C. For the temperature of 150 °C, this intensity is almost 80%, while 50% of the room temperature intensity is visible for the sample only at the temperature of 420 °C, i.e., 693 K (see Figures S4 and S5 in the Supplementary Materials). This proves the very good temperature stability of the measured sample.

**Figure 9:**
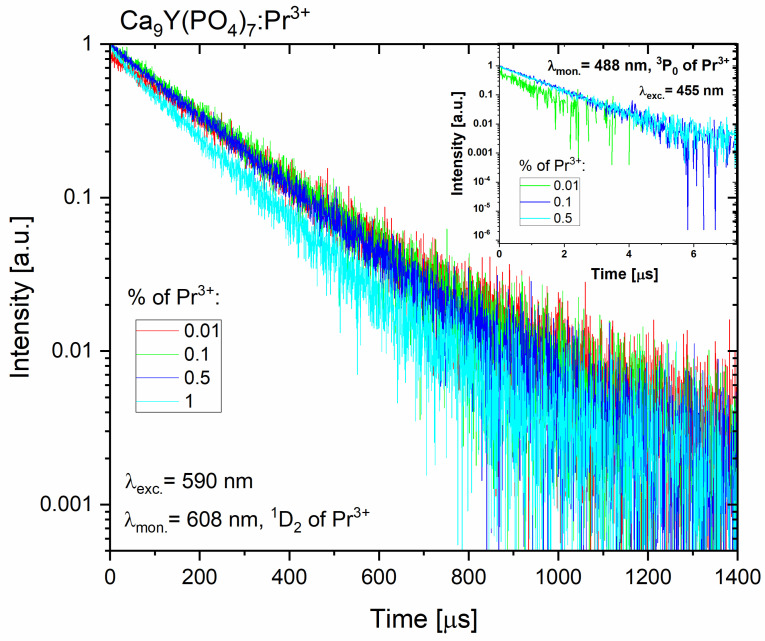
The luminescence decay curves of the emitting levels of Pr^3+^ ions in Ca_9_Y(PO_4_)_7_ polycrystals.

The luminescence decay times of the 4f emission are not monoexponential. However, the luminescence decay curve has a similar structure to a single-exponential function, which may indicate, on the one hand, the dominant influence of one of the sites (crystallographic positions), as well as the fact that the doped ions, located mainly in three different crystallographic positions, possess similar luminescence lifetimes (see Figure 9). The integral equation:

(4) $$\tau =\frac{\int I\cdot t\;dt}{\int I\;dt}$$

was used to calculate the mean value of the luminescence lifetime; thus, all components of the decay curve were averaged. The decay times of the luminescence from the ^1^D_2_ level to the ^3^H_4_ state are spin-forbidden; therefore, they are much longer than the decay times from the ^3^P_0_ level (see results in Table 2).

Concentration quenching is observed quite quickly for Pr^3+^ ions. A slight reduction in the luminescence decay time from the ^3^P_0_ level is visible from the concentration of 0.5% Pr^3+^, while a slight decrease in the length of the emission decay time for the ^1^D_2_ level begins quicker, where this effect is visible also for concentration lower than 0.5%, and it is very clear for the 1% of Pr^3+^. For the measured calcium phosphate polycrystals, the cross-relaxation process, as mentioned above, quenches the luminescence, and the multiphonon relaxation from the ^1^D_2_ level is less effective since the energy distance the lower ^1^G_4_ is above 7000 cm^−1^ and overt than five phonons must be involved.

## 3. Experimental

### 3.1. Synthesis

Ca_9_Y_1-x_Pr_x_(PO_4_)_7_ polycrystals with the concentration of Pr^3+^ (0.01; 0.1; 0.5; 1.0; 1.5; 2.0 mol%) were synthesized by solid-state reaction technique. The reagents CaCO_3_ (99.95%, Alfa Aesar, Haverhill, MA, USA), Y_2_O_3_ (99.99%, Acroc Organics, Geel, Belgium), Pr_2_O_3_ (99.9%, Chem PUR, Karlsruhe, Germany), NH_4_H_2_PO_4_ (99.999%, Acros Organics) were used as precursors. All precursors were weighed in the required proportion, placed in an agate mortar, and thoroughly ground. Then, annealing was carried out at a temperature of 700 °C for 2 h and then at a temperature of 1100 °C for 4 h. After each stage of annealing, the sample was ground again in an agate mortar.

### 3.2. Measurements

The X-ray powder diffraction (XRD) measurements were performed using an X’Pert PRO X-ray diffractometer (Panalytical, Malvern, UK). The reflectance absorption spectra were recorded using a Cary 5000 UV-VIS-NIR spectrophotometer. Pure Al_2_O_3_ powder was used as the baseline for spectrum correction. The excitation and emission spectra were measured using an FLS1000 fluorescence spectrometer from Edinburgh Instruments (Livingston, UK) equipped with a 450 W xenon lamp.

For the measurement of emission spectra, while using a xenon lamp with an excitation wavelength at 230 nm, the CYPO:Pr^3+^ polycrystalline powder was placed in a special holder with lithium fluoride (LiF) glass, which has high transparency in this area, and is better suited for measurements in the UVC range than a quartz tube, which may interfere with the measurement results of samples. The spectra taken using an FLS1000 spectrometer were automatically corrected to avoid the influence of the monochromator efficiency and the spectral response of the detector. For the temperature-dependent emission spectra, the Hamamatsu PMA-12 detector with Linkam THMS 600 Heating/Freezing Stage and laser diode at 450 nm were used. Decay time profiles were provided by using a Continuum Surelite I Optical Parametric Oscillator (OPO) pumped with a Nd:YAG laser. The UVC measurements were performed using a VUV McPherson spectrometer (McPherson, Chelmsford, MA, USA) equipped with a Czerny–Turner monochromator and a 150 W deuterium lamp. The upconversion emission was recorded on the McPherson spectrometer, using the excitation laser line at 444 nm focused on the area of 0.75 mm^2^ and corrected for the used filter characteristics.

The Raman spectrum was collected with a Renishaw inVia Raman spectrometer (Renishaw, Wotton-under-Edge, UK) equipped with a confocal DM2500 Leica optical microscope and an Ar-ion laser operating at 514.5 nm. The mid-IR ATR (attenuated total reflection) spectrum was measured using a Nicolet iS50 FT-IR spectrometer (Thermo Fisher Scientific, Waltham, MA, USA) equipped with an ATR accessory with a diamond crystal.

## 4. Conclusions

Ca_9_Y(PO_4_)_7_ has proven to be an excellent UVC phosphor when excited via the 5d bands, but it was also shown that despite the relatively short lifetime of the ^3^P_0_ level. It is likewise possible to obtain UVC emission when excited with blue light. According to our knowledge, this is the first paper describing the results of the UVC upconversion emission for this compound doped with the praseodymium(III) ions. The crystalline structures have been confirmed with the XRD measurement. The average crystallite sizes are in the nanoscale. Investigation in absorption, excitation, IR transmittance, Raman, and emission spectra, as well as the emission decay time profiles, were presented. Spectroscopic properties depend on the percent of doped rare-earth ions. The Pr^3+^ main luminescence of the 4f electronic configuration comes from the ^1^D_2_ multiplets. A shortening of luminescence decay times with increasing concentrations of Pr^3+^ doped ions was noticed. The emission wavelength range, predominantly falling within the UVC limit, coupled with the minimal emission intensity in the visible range upon direct excitation of the 4f^1^5d^1^ Pr^3+^ levels, renders this material a promising UVC phosphor. Consequently, further investigation into CYPO: Pr^3+^ as a scintillator for combating X-ray-resistant tumors is warranted. The future paths of our investigation will be focused on X-ray excitation of the modified CYPO host by exchange of Y with a heavier element like Lu, which will ensure a higher absorption cross-section.

However, the observed anti-Stokes emission in CYPO: Pr^3+^ appears to be less efficient compared to other established upconverters such as Y_2_SiO_5_: Pr^3+^, for example. It is possible that additional modifications to the synthesis and sample composition by exchange of the host cations may enhance the upconversion effect.

## Figures and Tables

**Table 1:** Lattice parameters, unit cell volume, and crystallite size of Ca_9_Y_1-x_Pr_x_(PO_4_)_7_ (0 ≤ x ≤ 0.02) phosphors calculated from XRD data.

	a (Å)	c (Å)	V (Å^3^)	Scherrer Method Size (nm)	W–H Plot Method	
					Size (nm)	Strain ×10^−3^
Ca_9_Y(PO_4_)_7_	10.4377	37.2636	3516.16	70	91	6.5
Ca_9_Y(PO_4_)_7_:0.01%Pr^3+^	10.4393	37.3020	3520.50	68	94	7.8
Ca_9_Y(PO_4_)_7_:0.1%Pr^3+^	10.4415	37.3116	3522.88	67	99	7.3
Ca_9_Y(PO_4_)_7_:0.5%Pr^3+^	10.4462	37.3248	3527.30	66	100	7.4
Ca_9_Y(PO_4_)_7_:1%Pr^3+^	10.4472	37.3656	3531.82	72	103	7.1
Ca_9_Y(PO_4_)_7_:1.5%Pr^3+^	10.4483	37.3706	3533.06	74	105	5.9
Ca_9_Y(PO_4_)_7_:2%Pr^3+^	10.4553	37.381	3538.76	77	106	5.4

**Table 2:** Luminescence lifetimes for Pr^3+^ ions in Ca_9_Y(PO_4_)_7_ matrices.

Sample	Lifetime [µs]	
	^3^P_0_ (Pr^3+^)	^1^D_2_ (Pr^3+^)
**Ca_9_Y(PO_4_)_7_:0.01% Pr^3+^**	1	211
**Ca_9_Y(PO_4_)_7_:0.1% Pr^3+^**	1	197
**Ca_9_Y(PO_4_)_7_:0.5% Pr^3+^**	0.9	187
**Ca_9_Y(PO_4_)_7_:1% Pr^3+^**	0.6	151

## Data Availability

Data are contained within the article.

## Supplementary Materials

The following supporting information can be downloaded at: https://www.mdpi.com/article/10.3390/molecules29092084/s1, Table S1: Distances between yttrium sites and oxygen ligands in Ca_9_Y(PO_4_)_7_; Figure S1: Plots of βcosθ versus 4sinθ for Ca_9_Y_1-x_Pr_x_(PO_4_)_7_ (0 ≤ x ≤ 0.02) nanoparticles; Figure S2: Absorption spectra of Ca_9_Y_0.085_Pr_0.015_(PO_4_)_7_; Figure S3: Excitation and emission spectra of Ca_9_Y_0.995_Pr_0.005_(PO_4_)_7_ polycrystals in the UVC range; Figure S4: Visualization of the temperature dependence luminescence intensity of Ca_9_Y_0.995_Pr_0.005_(PO_4_)_7_ for the selected temperatures; Figure S5: Quenching of the luminescence intensity of Ca_9_Y_0.995_Pr_0.005_(PO_4_)_7_ in a function of temperature.
