# Supplementary material for: UVC Stokes and Anti-Stokes Emission of Ca9Y(PO4)7 Polycrystals Doped with Pr3+ Ions
Source: Molecules. 2024 May 1;29(9):2084. doi: 10.3390/molecules29092084 (PMC11085382; doi:10.3390/molecules29092084)
Supplement: Supplementary file 1 [file molecules-29-02084-s001.zip › molecules-2918155-supplementary.pdf]

Supplementary information

UVC Stokes and anti-Stokes emission of $Ca_9Y(PO_4)_7$ polycrystals doped with Pr^{3+} ions

K. Lemański[1,*], O. Bezkrovna[1,2], N. Rebrova[1], R. Lisiecki[1], P. Zdeb[1], P. J. Dereń[1]

[1]Institute of Low Temperature and Structure Research, Polish Academy of Sciences, ul. Okólna 2, 50-422 Wrocław, Poland

[2]Institute for Single Crystals, NAS of Ukraine, Nauky Ave. 60, Kharkiv, 61001, Ukraine

Table S1. Distances between yttrium sites and oxygen ligands in $Ca_9Y(PO_4)_7$.

**Ca1|Y1 [Å] CN=8**	**Ca2|Y2 [Å] CN=8**	**Ca4|Y3 [Å] CN=6**
2.2751	2.2356	2.1478
2.4007	2.2700	2.1478
2.4124	2.3510	2.1478
2.4617	2.4110	2.2912
2.4342	2.4406	2.2912
2.5657	2.4595	2.2912
2.5612	2.6369	
2.7331	2.9940	
$Å_{av}$=2.4805	$Å_{av}$=2.4748	$Å_{av}$=2.2195

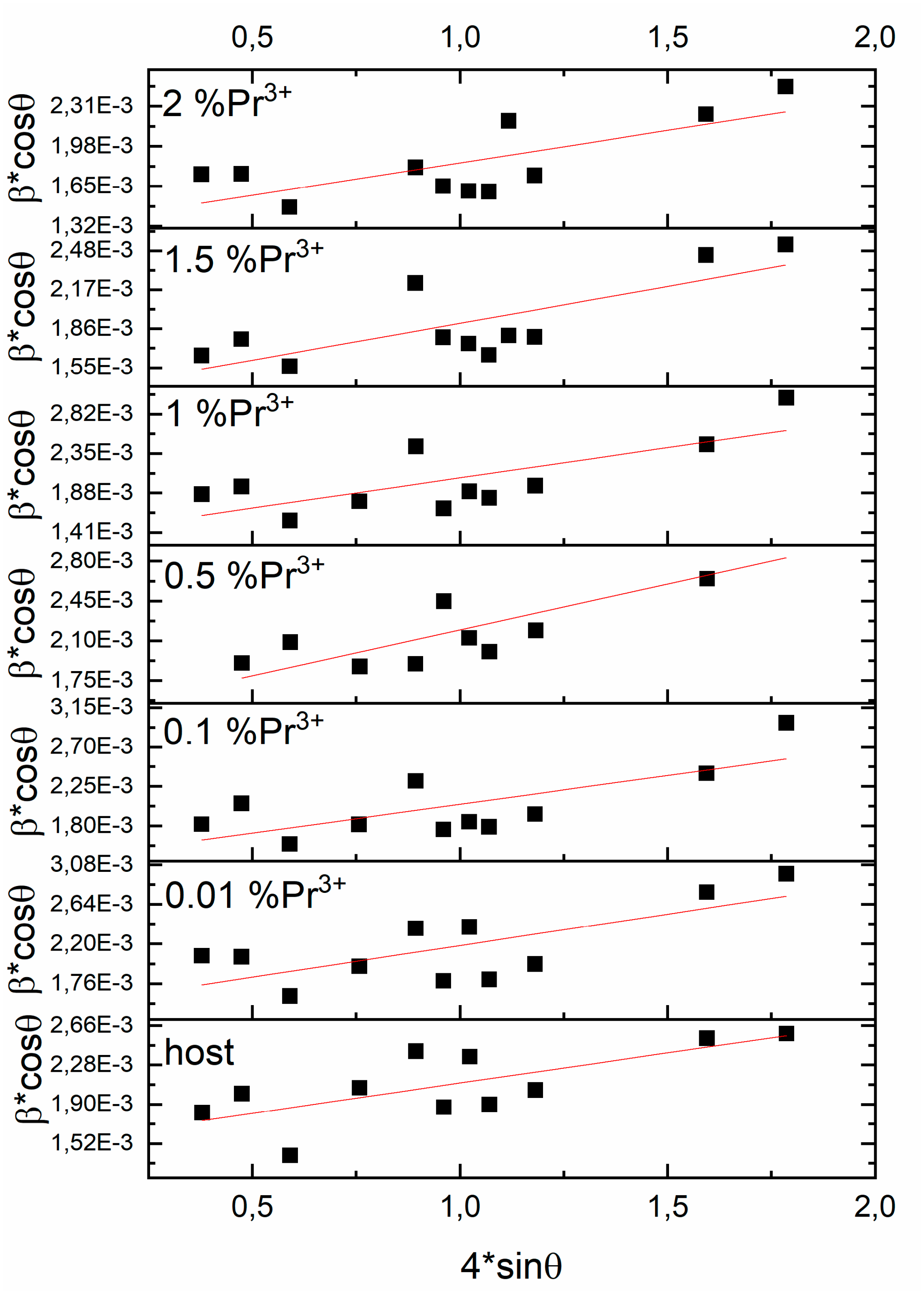

Figure S1. Plots of βcosθ versus 4sinθ for $Ca_9Y_{1-x}Pr_x(PO_4)_7$ (0≤x≤0.02) nanoparticles.

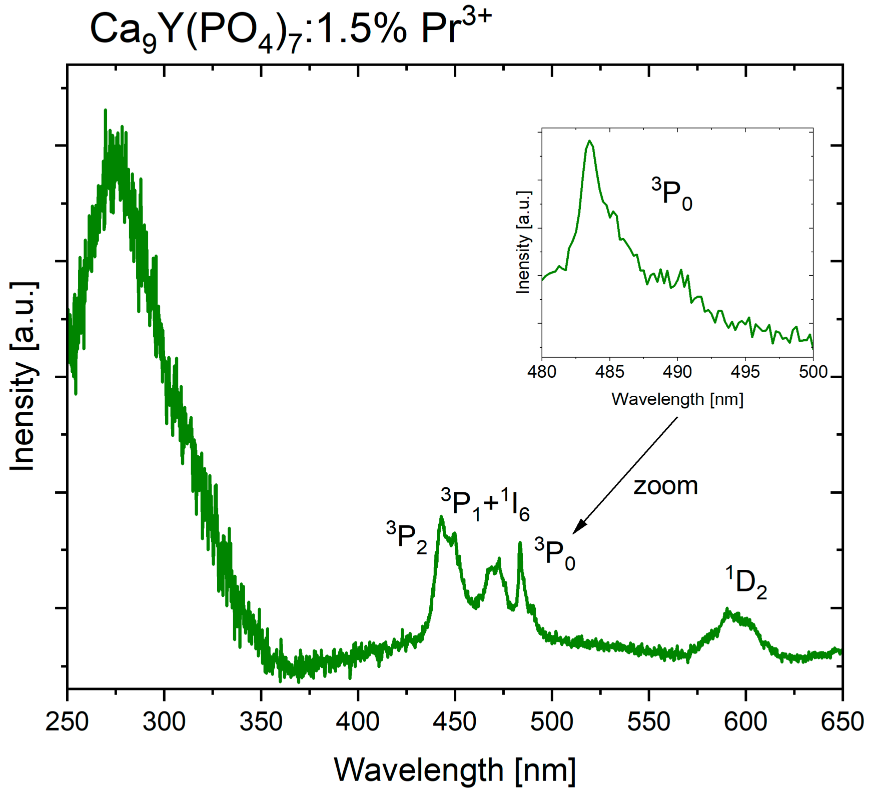

Figure S2. Absorption spectra of $Ca_9Y_{0.085}Pr_{0.015}(PO_4)_7$.

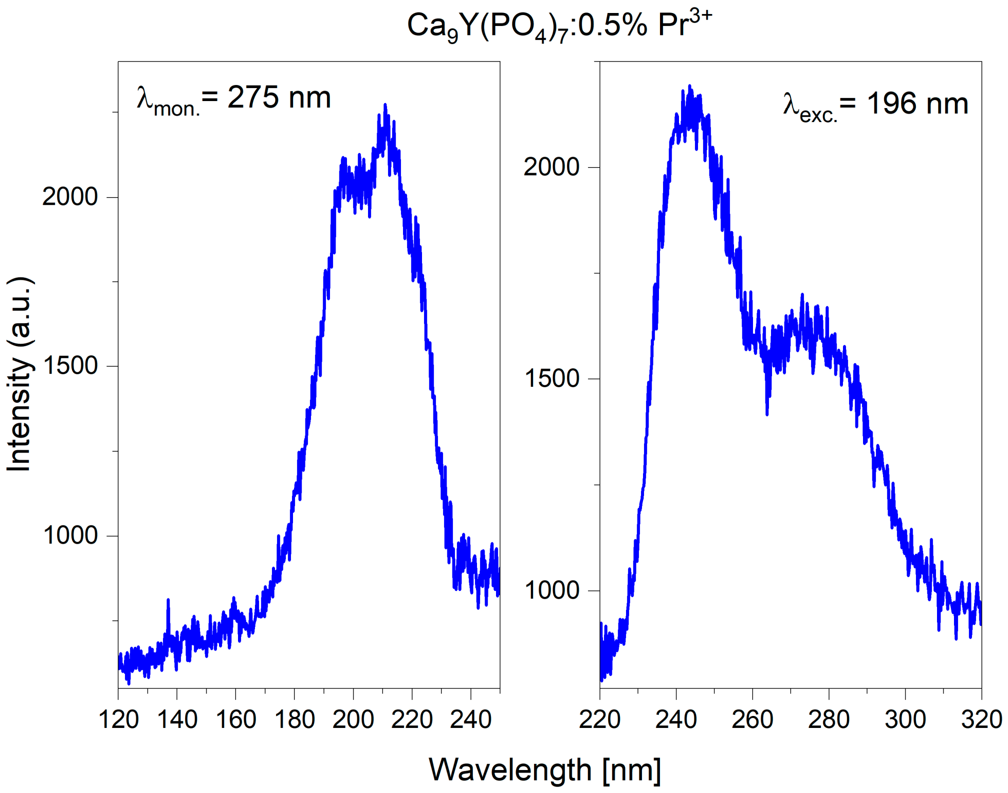

Figure S3. Excitation and emission spectra of $Ca_9Y_{0.995}Pr_{0.005}(PO_4)_7$ polycrystals in the UVC range.

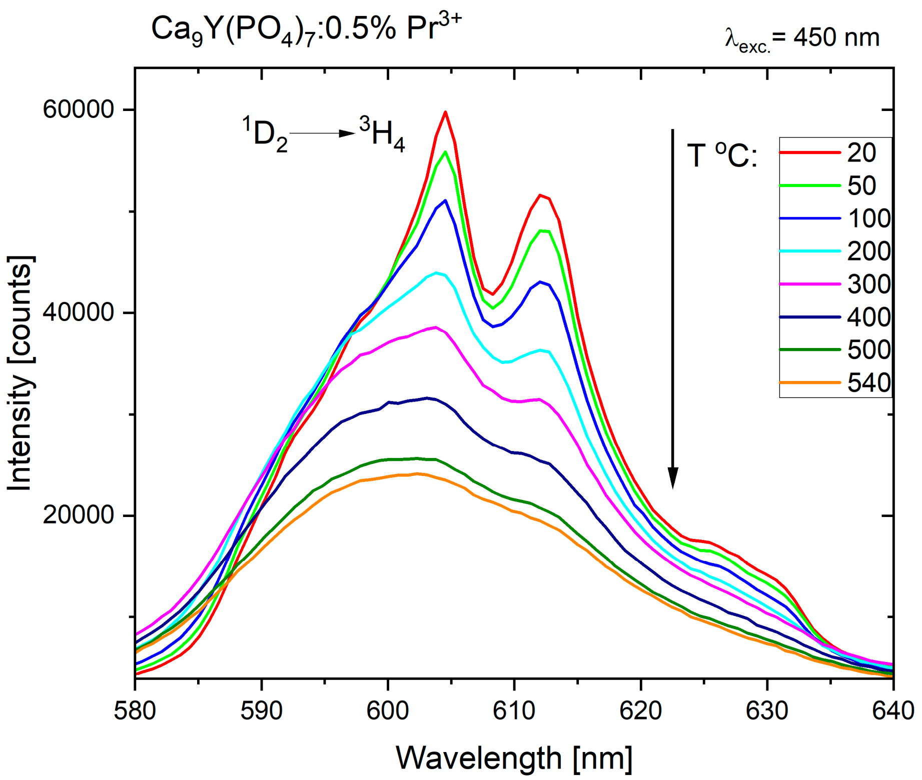

Figure S4. Visualization of the temperature dependence luminescence intensity of $Ca_9Y_{0.995}Pr_{0.005}(PO_4)_7$ for the selected temperatures.

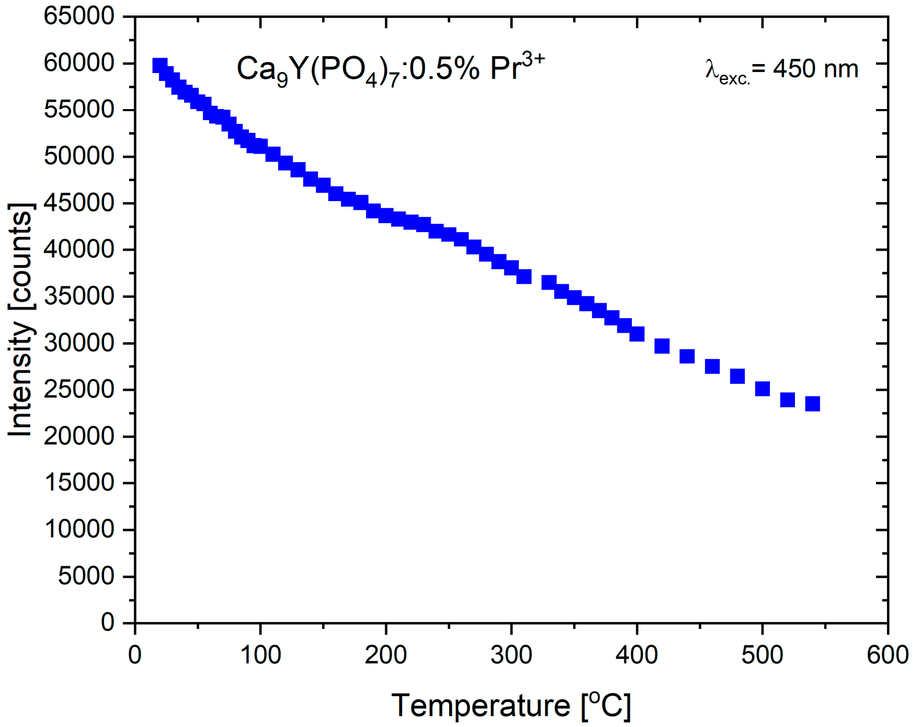

Figure S5. Quenching of the luminescence intensity of $Ca_9Y_{0.995}Pr_{0.005}(PO_4)_7$ in a function of temperature.
